# Supplementary material for: Unconventional Hall effect and its variation with Co-doping in van der Waals Fe3GeTe2
Source: Sci Rep. 2021 Jul 8;11:14121. doi: 10.1038/s41598-021-93402-6 (PMC8266818; doi:10.1038/s41598-021-93402-6)
Supplement: Supplementary file 1 — Supplementary Information. [file 41598_2021_93402_MOESM1_ESM.pdf]

Unconventional Hall effect and its variation with Co-doping in van der Waals Fe_3GeTe_2

Rajeswari Roy Chowdhury[1*], Samik DuttaGupta[2-4*], Chandan Patra[1], Oleg A. Tretiakov[5], Sudarshan Sharma[1], Shunsuke Fukami[2-4,6,7], Hideo Ohno[2-4,6,7] and Ravi Prakash Singh[1*]

[1] Department of Physics, Indian Institute of Science Education and Research Bhopal, Bhopal Bypass Road, Bhauri, Madhya Pradesh 462-066, India
[2] Center for Science and Innovation in Spintronics, Tohoku University, 2-1-1 Katahira, Aoba-ku, Sendai 980-8577, Japan
[3] Center for Spintronics Research Network, Tohoku University, 2-1-1 Katahira, Aoba-ku, Sendai 980-8577, Japan
[4] Laboratory for Nanoelectronics and Spintronics, Research Institute of Electrical Communication, Tohoku University, 2-1-1 Katahira, Aoba-ku, Sendai 980-8577, Japan
[5] School of Physics, The University of New South Wales, Sydney 2052, Australia
[6] Center for Innovative Integrated Electronic Systems, Tohoku University, 468-1 Aramaki Aza Aoba, Aoba-ku, Sendai 980-0845, Japan
[7] WPI Advanced Institute for Materials Research, Tohoku University, 2-1-1 Katahira, Aoba-ku, Sendai 980-8577, Japan
**Corresponding author:* rajeswari@iiserb.ac.in, sdg@riec.tohoku.ac.jp, rpsingh@iiserb.ac.in

SUPPLEMENTARY INFORMATION

S1. Determination of Curie temperature for samples with/without Co-doping

S2. Stability of Co-doping in ambient conditions

S3. Determination of first and second-order magnetic anisotropy constants

S4. Reproducibility of Hall resistivity (ρ_{XY}) versus H_X behavior

S5. Hall resistivity (ρ_{XY}) versus $H_{X,Y}$

S6. Determination of material-specific factor (S_A)

S1. Determination of Curie temperature for samples with/without Co-doping

We evaluate the Curie temperature of our single-crystalline samples by calculating the derivative of magnetic susceptibility (χ) from the temperature-dependent magnetization measurements. Figure S1(a)-(d) shows the plot of derivative of χ versus temperature (T) under applied magnetic field H (both for H || c-axis and $H \perp c$-axis) for FGT, $Co_{0.05}$FGT, $Co_{0.45}$FGT, and $Co_{0.55}$FGT, respectively. The position of the minimum in dχ/dT corresponds to the ferromagnetic transition temperature or Curie temperature (T_C) in our single-crystalline samples. We obtain a reasonable agreement of T_C determined from magnetization (M)-T curves with applied H || c-axis and ⊥ c-axis.

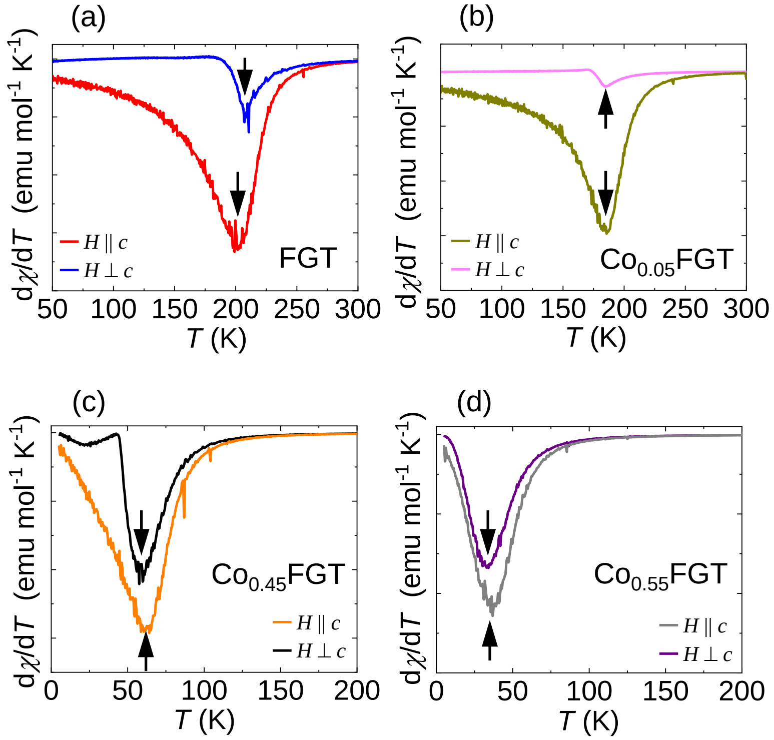

Figure S1. Derivative of magnetic susceptibility (dχ/dT) versus temperature (T), calculated from the temperature dependence of magnetization (M-T) measurements, for both configurations of applied H (H || c-axis or H). The black arrows in (a)-(d) denote the Curie temperature obtained as a minimum of dχ/dT-T curves.

S2. Stability of Co-doping in ambient conditions

In this study, bulk FGT and Co_xFGT (x = 0.05, 0.45, 0.55) were grown by chemical vapor transport (CVT) technique showing near-perfect crystalline and ferromagnetic behavior, consistent with most of the previous reports [R1-R3]. According to a previous study [R4], ambient oxidation of FGT can occur over several weeks, forming an amorphous antiferromagnetic oxidized-FGT top layer. This surface oxidation results in a significant variation of magnetization (*M*) versus applied *H* (|| *c*-axis) curves and associated reduction of the anomalous Hall resistivity magnitude. To check the possibility of oxidation behavior, we carried out *M*-*H* measurements on our Co-doped single-crystals immediately after synthesis (pristine state) and after keeping them in ambient condition for several weeks (the samples were wrapped using Teflon tape and preserved in ambient conditions for more than three weeks). Figure S2 shows the experimental results of such measurements on $Co_{0.05}$FGT (at T = 150 K), $Co_{0.45}$FGT (at T = 50 K), and $Co_{0.55}$FGT (at T = 50 K). Our experimental results do not show any noticeable changes in the magnitude or shape of the magnetization curves, indicating the stable behavior with Co-doping.

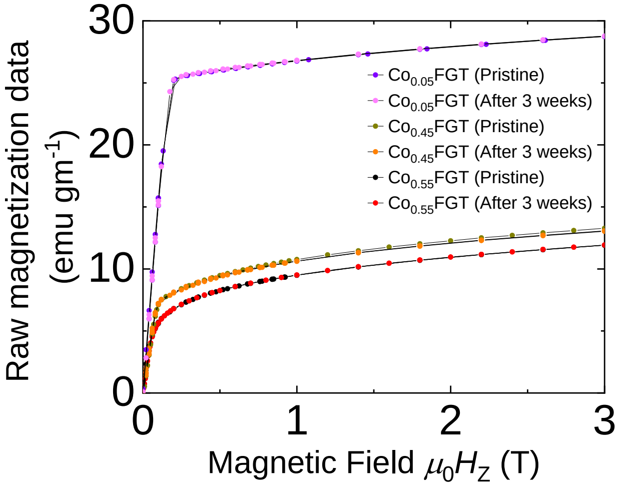

Figure S2. Experimental results of magnetization (*M*) versus applied magnetic field (*H*) (|| *c*-axis) for $Co_{0.05}$FGT (at T = 150 K), $Co_{0.45}$FGT (at T = 50 K), and $Co_{0.55}$FGT (at T = 50 K). The pristine state corresponds to the measurements carried out immediately after the synthesis of the crystal, while the subsequent measurements on the same crystal were carried out after more than three weeks.

S3. Determination of first and second-order magnetic anisotropy constants

For a uniaxial ferromagnetic system, the total magnetic free energy (***E***) of the system is given by

$$\boldsymbol{E} = K_1 \, \mathrm{Sin}^2\theta + K_2 \, \mathrm{Sin}^4\theta - HM_S \, \mathrm{Cos}\theta \tag{1}$$

where K_1, K_2 are the first and second-order magnetic anisotropy constants, respectively, H is the applied external magnetic field, M_S is the saturation magnetization, and θ corresponds to the angle between the magnetization direction and the magnetic easy-axis (*i.e.*, *c*-axis for our case). According to Sucksmith Thompson's model [R5], for small applied H, perpendicular to the easy-axis ($H \parallel ab$-plane)

$$\mathrm{Sin}\theta \approx \frac{M}{M_S} \tag{2}$$

where M is the magnetization value for applied $H \perp c$-axis. On minimizing ***E*** using Eqn. (2), we arrive at the following relation describing the magnetization (M-H) curve with $H \perp c$-axis

$$\frac{H}{M} = \frac{4K_2}{M_S^4} M^2 + \frac{2K_1}{M_S^2} \tag{3}$$

Thus, from the fitting of the H/M versus M^2 curves with H ⊥ *c*-axis, K_1 and K_2 can be determined from the slope and intercept of the linear fit. Figure S3 (a), (b) shows the experimental results and the linear fitting for $Co_{0.55}$FGT and $Co_{0.45}$FGT single-crystalline samples at $T = 50$ K. From the slope and intercept, we obtain $K_1 = 4.18 \times 10^5$, $K_2 = 0.58 \times 10^5$ J m^{-3} for $Co_{0.05}$FGT, and $K_1 = 3.19 \times 10^4$ J m^{-3}, $K_2 = 0.42 \times 10^4$ J m^{-3} for $Co_{0.45}$FGT.

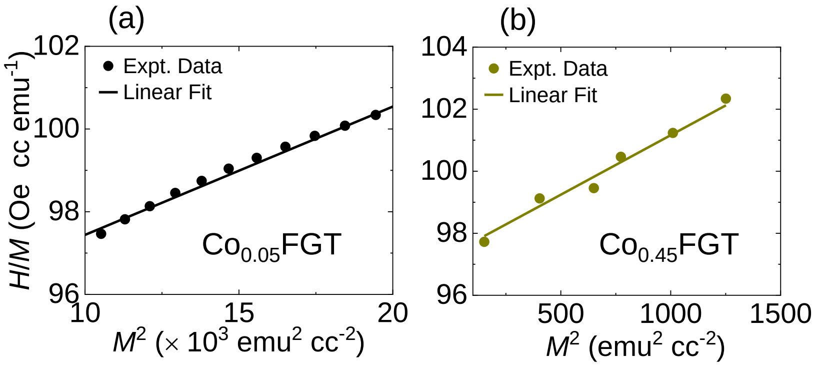

Figure S3. H/M versus M^2 curves for $Co_{0.05}$FGT and $Co_{0.45}$FGT at $T = 50$ K. Black and green circles in (a) and (b) denote experimental data from M-H curves with $H \perp c$-axis. Continuous lines in (a), (b) indicate linear fit with Eq. (3).

S4. Reproducibility of Hall resistivity (ρ_{XY}) versus H_X behavior

To eliminate any possible sample-dependent effects giving rise to the unconventional Hall effect in Hall resistivity (ρ_{XY}) versus H_X, we carried out magnetotransport measurements on different single crystals (with/without Co-doping) prepared on different runs (series 1-4). Figure S4 (a), (b) shows the experimental results of ρ_{XY} versus H_X for two different $Co_{0.05}$FGT (series 1 and 2) and $Co_{0.45}$FGT (series 3 and 4) single-crystalline samples, respectively, synthesized on separate runs. Our experimental results show a minuscule variation of the ρ_{XY} magnitude and the corresponding magnetic field magnitude leading to a cusp-like maximum in ρ_{XY}, for the samples of different series at a fixed Co-doping level. These experimental results rule out any contribution of extrinsic effects (such as sample-to-sample variations) to the observed unconventional Hall effect behavior.

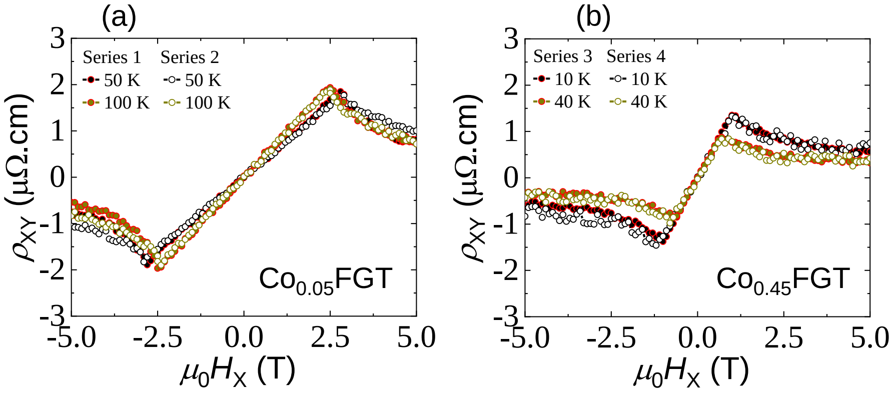

Figure S4. (a) Hall resistivity (ρ_{XY}) versus H_X (⊥ c-axis || I) for two separately prepared (series 1 and series 2) $Co_{0.05}$FGT single-crystalline samples at 50 K and 100 K. (b) Experimental results for similar measurements on separately prepared (series 3 and series 4) $Co_{0.45}$FGT single crystalline samples at 10 K and 40 K.

S5. Hall resistivity (ρ_{XY}) versus $H_{X,Y}$

To investigate the existence of possible variations of the unconventional Hall effect (described in the main text) and the strength of the emergent magnetic field along different in-plane directions, we have measured Hall resistivity (ρ_{XY}) by sweeping the magnetic field along x and y directions. Figure S5 shows the experimental results of one such measurement for the FGT single crystal. The experimental configuration is the same as mentioned in the main text. Our results show that the magnitude of ρ_{XY} and the peak position is independent of the direction of the applied magnetic field along any in-plane directions.

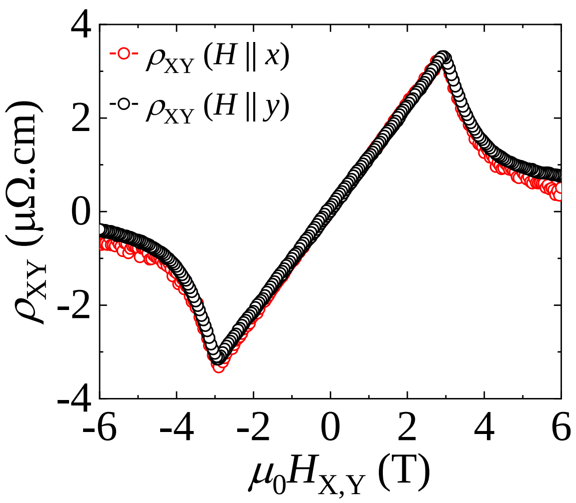

Figure S5. Hall resistivity (ρ_{XY}) versus H_X or H_Y ($\perp$ c $\parallel$ I) for the FGT single-crystalline samples at 100 K.

S6. Determination of material-specific factor (S_A)

The Hall resistivity (ρ_{XY}) of an FM is empirically expressed as the sum of the ordinary and anomalous Hall terms. The anomalous Hall coefficient is usually dependent on both the magnetization and longitudinal resistivity (ρ_{XX}) and is field-dependent. Taking into account the field dependence of resistivity, the Hall resistivity in the absence of additional magnetoresistive contributions can be written as [R5, R6]

$$\rho_{XY} = \mu_0 R_0 H + S_A \rho_{XX}^2 M, \tag{1}$$

where ρ_{XX} is the longitudinal resistivity of the single crystal, S_A is the field independent coefficient to the anomalous Hall resistivity [R3], and R_0 is the ordinary Hall coefficient. Thus, S_A can be determined as the intercept of ($\rho_{XY}/\mu_0 H$) versus ($\rho_{XX}^2 M/\mu_0 H$) curves. Figure S6 shows the experimental results and linear fitting for Co-FGT and FGT at 100 K. The obtained values of S_A at each temperature were utilized to extract the topological Hall contribution from the Hall resistivity.

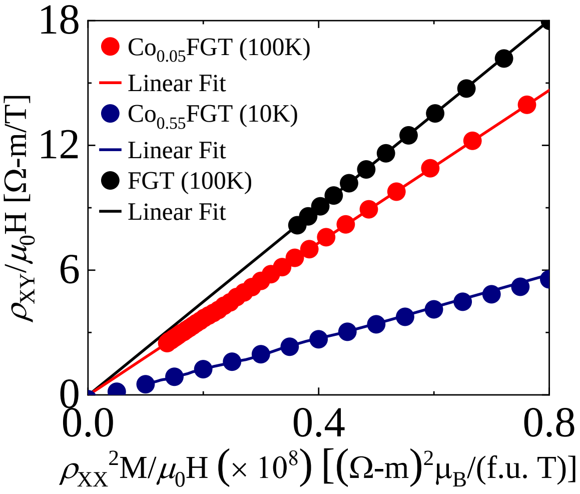

Figure S6. $\rho_{XY}/\mu_0 H$ versus $\rho_{XX}^2 M/\mu_0 H$ (H ⊥ *c*-axis) for the Co_xFGT and FGT single-crystalline samples at 100 K.

References

[R1] Wang, Y. *et al*. Phys. Rev. B. **96**, 134428 (2017).
[R2] Kim, K. *et al*. Nature Mater. **17**, 794 (2018).
[R3] Tian, C.-K. *et al*. Phys. Rev. B. **99**, 184428 (2019).
[R4] Kim, D. *et al.* Nanotechnology **30**, 245701 (2019).
[R5] Sucksmith, W. & Thompson, J. E. Proc. R. Soc. London, Ser. A **225**, 362 (1954).
[R4] Kanazawa, N. *et al*. Phys. Rev. Lett. **106**, 156603 (2011).
[R5] Wang, Y. *et al.* Phys. Rev. B **96**, 134428 (2017).
[R6] Lee, M. *et al*. Phys. Rev. B **75**, 172403 (2007).
